# Computational Analysis Reveals the Association of Threonine 118 Methionine Mutation in PMP22 Resulting in CMT-1A

**DOI:** 10.1155/2014/502618

**Published:** 2014-10-20

**Authors:** Chundi Vinay Kumar, Rayapadi G. Swetha, Anand Anbarasu, Sudha Ramaiah

**Affiliations:** ^1^School of Biosciences and Technology, VIT University, Vellore, Tamil Nadu 632014, India; ^2^Bioinformatics Division, School of Biosciences & Technology (SBST), VIT University, Vellore 632014, India

## Abstract

The T118M mutation in PMP22 gene is associated with Charcot Marie Tooth, type 1A (CMT1A). CMT1A is a form of Charcot-Marie-Tooth disease, the most common inherited disorder of the peripheral nervous system. Mutations in CMT related disorder are seen to increase the stability of the protein resulting in the diseased state. We performed SNP analysis for all the nsSNPs of PMP22 protein and carried out molecular dynamics simulation for T118M mutation to compare the stability difference between the wild type protein structure and the mutant protein structure. The mutation T118M resulted in the overall increase in the stability of the mutant protein. The superimposed structure shows marked structural variation between the wild type and the mutant protein structures.

## 1. Introduction

Charcot-Marie-Tooth (CMT) neuropathies are traditionally called hereditary motor and sensory neuropathies (HMSNs) [1]. CMT has two main groups, namely, CMT disease type 1 (CMT1) and CMT disease type 2 (CMT2). Charcot-Marie-Tooth disease is categorized into two main groups on the basis of electrophysiological properties and histopathology: primary peripheral demyelinating neuropathy or CMT1 and primary peripheral axonal neuropathy or CMT2. Neuropathies of CMT1 group are characterized by strictly reduced nerve conduction paces (less than 38 m/sec), segmental demyelination, and remyelination with onion bulb formations on nerve biopsy, slowly progressive distal muscle atrophy and weakness, absent deep tendon reflexes, and hollow feet. CMT1A inheritance is autosomal dominant [2] and in CMT type 2 the NCVs tend to remain normal but there is a reduction in the conduction amplitudes. One per 2,500 individuals has the clinical phenotype of CMT which is a very common disorder in humans that is inherited [1]. The mutation in peripheral myelin protein 22 (PMP22) gene that is mapped to 17p12-p11.2 is seen to be associated with Charcot Marie Tooth, type 1A (CMT1A) [3–5]. CMT1A is a form of Charcot-Marie-Tooth disease, the most common inherited disorder of the peripheral nervous system [4]. The average onset age of CMT1A patients is seen to be +/− 7.3 of 12.2 years. The diagnostics of CMT1A patients show nerve conduction velocity to be as slow as 38 m/s [3, 6]. Pmp22 gene encodes a specific integral membrane protein which is a major component of myelin in peripheral nervous system [7]. The PMP22 gene has a causative role in CMT type 1. One or the other point mutation in PMP22 or a duplication of the region including PMP22 gene can result in the disease phenotype [8]. Missense mutations in PMP22 cause variable syndromes similar to that found with HNPP (heredity neuropathy with liability to pressure plates), suggesting that they yield a loss of PMP22 function [9]. The bulk of PMP22 missense mutations, on the other hand, act genetically as autosomal dominants, proposing that they harvest a gain of PMP22 function. A few alterations, such as R157W, R157G, and T118M, are autosomal recessive in nature [10]. A missense mutation at codon 118 of PMP22 gene fabricating a threonine to methionine amino acid substitution (T118M) has been testified in the context of familial neuropathy; however, both its clinical importance and its influence on PMP22 function are divisive. The mutation was originally recognized in a female with severe CMT1 who was hemizygous for T118M because of a HNPP deletion at the other allele [11]. Her two sons who inbred the HNPP deletion had a distinctive HNPP phenotype, while the third son who inbred T118M mutation was normal, suggesting that T118 mutation is recessive. Successively, numerous investigators identified the T118M mutation in unaffected parents of neuropathy patients or in compound heterozygotes whose phenotype was not different from either HNPP of CMT1A. The T118M mutation has also been stated in a family in which the parents had a slight phenotype and carried both a CMT1A duplication and T118M mutation, whereas a younger family member had a more severe disease but carried only the duplication, telling that T118M is a part loss-of-function mutation which can mitigate the effects of the duplication [11]. Later the mutation T118M of the PMP22 gene was seen to be a causative mutation in many CMT1A cases [8, 11–13].

Nonsynonymous single nucleotide polymorphisms (nsSNPs) are generally identified to have the potential to alter the way the corresponding protein functions, either directly or via disruption of the structures [14]. In NCBI dbSNP database [15], there are about 41,744,328 human SNPs that have been validated and submitted.

Making use of computational platforms for the prediction of disease-associated nsSNPs has become a very common methodology. The identification of the deleterious and disease-related mutations was initiated by several research articles, hence predicting the pathogenic nsSNPs in correlation with their functional and structural damaging properties [16–18]. In this study our main focus is to prioritize the pathogenic alleles in the PMP22 gene and their structural consequence at molecular level. Various tools such as SIFT [19], PolyPhen2 [20], PMUT [21], SNP&GO [22], PhD-SNP [23], and MutPred [24] are used for prioritizing the deleterious disease-associated nsSNPs from the obtained SNP datasets from the NCBI dbSNP database. The alleles with very high structural damaging probability can possibly lead to a major loss of PMP22 functionality. SNPs which showed positive implication of pathogenicity by all the used tools were considered as strong candidates for structural analysis. SNPs with intermediate pathogenicity implications were taken out from the study.

Molecular dynamics simulation of the wild type PMP22 protein and the most deleterious mutant to examine the molecular and structural basis of predicted disease-associated nsSNPs is performed. For the protein trajectories and atomic interaction analysis, g_rms, g_rmsf, g_hbond, g_sas, and g_gyrate, GROMACS inbuilt tools were used successfully. We used g covar and g anaeig modules of GROMACS package to conduct the principle component analysis (PCA) and estimate the flexibility of the structure.

## 2. Materials and Methods

### 2.1. Dataset

Data on human PMP22 gene was collected from OMIM [25] and Entrez gene on National Center for Biotechnology Information (NCBI) website. The SNP information of PMP22 was got from the BioMed Research International 3 dbSNP database [15]. The amino acid sequence of this protein was taken from the UniProt database (UniProt ID: Q01453).

### 2.2. Disease-Associated SNP Prediction

The presence of single nucleotide polymorphism may lead to the deleterious consequence in its 3D structures and hence may lead to disease-associated phenomena. We used SIFT [19], PolyPhen2 [20], PMUT [21], SNP&GO [22], PhD-SNP [23], and MutPred [24] to examine the disease-associated nsSNP in the PMP22 protein coding region.

Homology-based approach was made use of by the SIFT server to classify amino acid substitutions. In the SIFT server, if the prediction score of the mutation was >0.05 then that particular mutation was considered to be deleterious and if the prediction score of the mutation was <0.05 the mutation is considered to be tolerated [19]. The combination of sequence and structure-based attributes is the base of PolyPhen 2.0 server and this server generally uses naïve Bayesian classifier for the identification of an amino acid substitution and the impact of mutation. The PolyPhen 2.0 server classifies the output levels as probably damaging and possibly damaging which are done as functionally significant (≥0.05) and benign level being classified as tolerated (≤0.05) [20]. PMUT is a neural network-based program that is trained on large database of neutral and pathological mutations. Three different parameters are used in PMUT that include mutation descriptors, solvent accessibility, and residue and sequence properties. These properties are used to calculate the pathogenicity indexes of the given mutations ranging from 0 to 1. The mutations with index score greater than 0.5 are predicted to be pathologically significant [21].

The nsSNPs of the PMP22 gene that were commonly predicted to be deleterious and damaging from these three servers were considered for further analysis. We further made use of SNP&GO, PhD-SNP, and MutPred tools to examine the disease-associated nsSNPs. The data retrieval sources for SNP&GO include protein sequence, evolutionary information, and functions as encoded in the gene ontology terms [22]. PhD-SNP is a SVM based classifier, trained over the million amino acid polymorphism datasets making use of the supervised training algorithm. It predicts whether the given amino acid substitution is disease associated or neutral along with the reliability index score [23]. MutPred was commonly used as a web based tool. It was used with the main focus of predicting the molecular changes associated with amino acid variants. MutPred uses SIFT, PSI-BLAST, and Pfam profiles along with some structural disorder prediction algorithms, including TMHMM, MARCOIL, I-Mutant 2.0, B-factor prediction, and DisProt. On combining the results and prediction of all the six servers, the accuracy of prediction rises to a greater extent and finally the most disease-associated mutations are filtered.

### 2.3. Molecular Dynamics and Simulation

The simulation of the wild type and mutant PMP22 proteins was performed using GROMACS 4.5.5 software [26]. The force field used for simulation is Gromos96 53a6 [27, 28]. The structures were solvated using the simple-point-charge (SPC) water box with dimension of 52.0 Å with molecules of water. At physiological pH protein was charged positively; hence, to make the simulation system electrically neutral, the system was neutralized by adding counter ions (Cl^−^ or Na^+^). Steepest descent method was used to do energy minimization for 1000 steps. After minimization, three different steps were used in the MD simulation, namely, heating, equilibration, and production. NVT ensemble (constant number of particles, volume, and temperature) was used (300 K and 1.0 atm) [29] followed by the NPT ensemble (constant number of particles, pressure, and temperature) which was performed for 1000 ps at 300 K. The production simulation was carried out at 300 K for 50 ns wild type and mutant of PMP22 protein. All the covalent bonds were constrained by using the LINCS (Linear Constraint Solver) algorithm [30]. The electrostatic interactions were treated using Particle Mesh Ewald (PME) method [31]. The cutoff radii for coulomb and van der Waals interactions were set to 10.0 and 14.0 Å, respectively. The MD trajectories, which were saved every 2.0 ps, were analyzed using GROMACS.

The potential of each trajectory produced was thoroughly analyzed after MD simulations. The MD trajectories were analyzed using g_rms, g_rmsf, g_hbond, and g_gyrate of GROMACS utilities [32] to get the root-mean-square deviation (RMSD), root-mean-square fluctuation (RMSF), radius of gyration (Rg), and the number of H bonds formed between the ligand and proteins. The differences in kinetic, potential, and total energies, pressure, and temperature were computed as a function of simulation time to see whether the systems obey NVT or NPT ensemble throughout the simulation. The total number of hydrogen bonds was calculated to understand the difference in ligand-protein stability. SASA was performed to understand the solvent accessible surface area. The trajectories were analyzed by using the tools from GROMACS distribution. All the graphs were generated using the XMgrace tool [33]. Essential dynamics (ED) [34] was performed for all the trajectories according to principal component analysis (PCA). The first two eigenvectors (principal components PC1 and PC2) with largest eigenvalues were used to make 2D projection for each of independent trajectories. For the simulation of both wild type and mutant PMP22, C*α* atoms were included in the definition of the covariance matrices for the protein. Both the protein structures were subjected to online tools to predict protein structures.

## 3. Results

### 3.1. Protein Modeling

**Figure 1:**
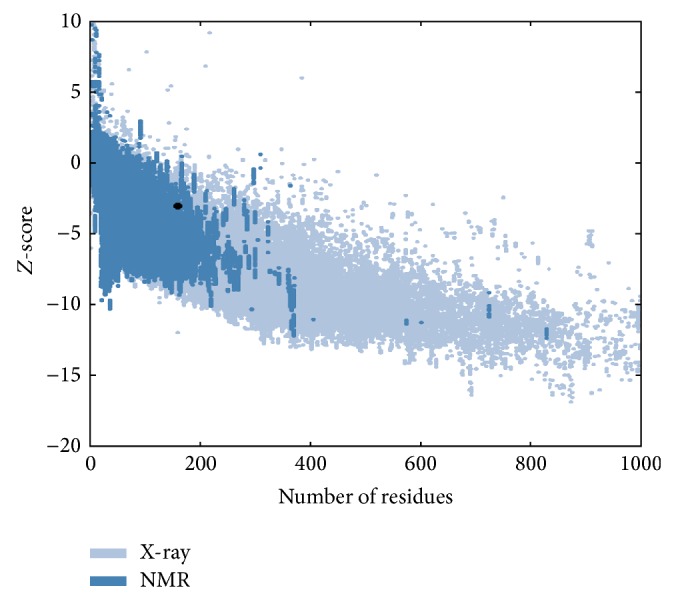
Protein structure validation using ProSA server.

The PMP22 protein structure was modeled using I-tasser [35, 36]. The structure validation of modeled protein was performed (Figure 1) and the results suggest that the modeled PMP22 protein has the quality of NMR structures.

### 3.2. Prediction of Deleterious nsSNPs Using SIFT, PolyPhen2, and PMUT Programs

Out of 26 input polymorphic datasets, 20 nsSNPs are found to be “damaging” (0.5 to 1.000) to protein structure and function and the remaining 6 nsSNPs are characterized as benign by PolyPhen 2.0. Among these 20 deleterious nsSNPs, 8 SNPs are reported to be highly deleterious with PolyPhen2 score of 1.000 (Table 1). In SIFT, 13 mutations are predicted to be deleterious with tolerance index ≥ 0.05 (Table 1). Among these, 6 mutations (R157W, S72L, A67T, S22F, W28R, and Y136S) are reported to be highly deleterious with SIFT score of 0.00 (Table 1). Furthermore, 13 mutations are identified as deleterious and damaging in SIFT and PolyPhen 2.0 server (Table 1) which also showed a very strong correlation between the prediction methodologies implemented by these two servers. SIFT and PolyPhen2 are seen to have better performance in identifying functional nsSNPs among other* in silico* tools [37]. The accuracy of SIFT and PolyPhen2 is further validated through our results, which makes these tools more suitable for the prediction. All the nsSNPs submitted to PolyPhen 2.0 and SIFT are also analyzed using PMUT server. 15 mutations are predicted to cause pathological effect by PMUT. The remaining 11 mutations show neutral effect. From the 26 input polymorphic datasets, we filtered 11 (R157W, L16P, S79C, T118M, M69K, H12Q, G150C, S22F, W28R, D37V, and Y136S) mutations which are predicted to be deleterious as well as damaging using SIFT, PolyPhen2, and PMUT servers (Table 1).

### 3.3. Prediction of Disease-Associated nsSNPs

Total 11 nsSNPs are commonly predicted in SIFT, PolyPhen 2.0, and PMUT. To these 11 mutations, we applied PhD-SNP which is based on support vector machine tool to further classify the predicted deleterious nsSNPs as disease associated. In the PhD-SNP server, all 11 mutations (R157W, L16P, S79C, T118M, M69K, H12Q, G150C, S22F, W28R, D37V, and Y136S) are predicted to be disease associated (Table 2). In SNP&GO, 10 (R157W, L16P, T118M, M69K, H12Q, G150C, S22F, W28R, D37V, and Y136S) nsSNPs are predicted to be disease associated (Table 2). Overall, 10 mutations (R157W, L16P, T118M, M69K, H12Q, G150C, S22F, W28R, D37V, and Y136S) are predicted as most disease-associated mutations by both PhD-SNP and SNP&GO (Table 2). These 10 mutations are further analyzed by MutPred tool to predict the SNP disease-association probability and probable change in the molecular mechanism in the mutant. We found T118M to be highly deleterious with general probability (*g*) scores of 0.948 and it is predicted to induce the loss of sheet at T118 with (*p*) score of 0.0457, showing high confidence hypothesis. Finally, we screened T118M as the most deleterious and disease-associated mutation in PMP22 gene (Table 3). This prediction could be endorsed with the noticed experimental data [8, 11–13]. We explored T118M mutation in detail.

### 3.4. RMSD

The MD simulation resulted in the generation of various plots. One among them is the RMSD plot. The RMSD backbone value for the wild type protein structure and the mutant is calculated against the time simulation between 0 and 50000 ps. The RMSD is a crucial parameter to analyze the equilibration of MD trajectories. It is estimated for backbone atoms by using the wild type and mutant structure of the MD simulations. The RMSD of the backbone atoms relative to the corresponding starting structures are calculated.

#### 3.4.1. RMSD for Wild Type Protein Structure

**Figure 2:**
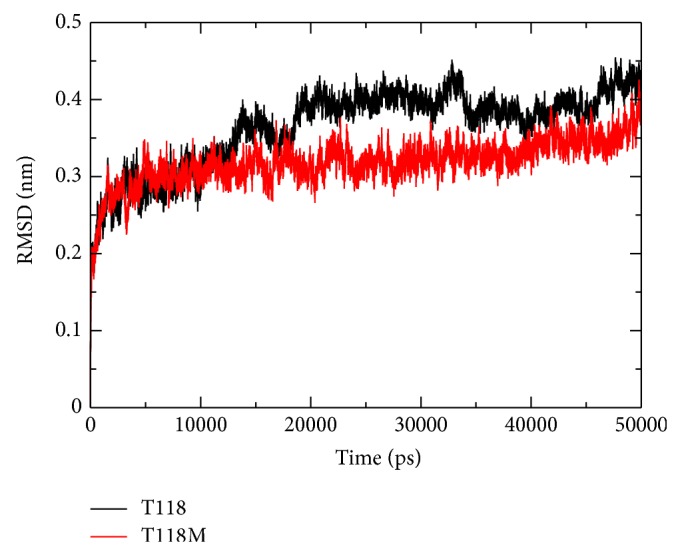
Backbone RMSD are shown as a function of time for wild type (black) and mutant (red) PMP22 protein motor domain structures at 300 K.

In the wild type protein structure we can notice that the average RMSD trajectory value ranges between 0.17 nm and 0.45 nm. This protein structure shows deviations throughout the simulation time period. After 18500 ps increase in the RMSD trajectory value of the wild type protein structure to 0.45 nm is clearly observed. After 34200 ps the RMSD value of this structure is seen to drop to 0.37 nm. A slight raise of RMSD value can be noticed at 46000 ps after which it is constant till the end. The RMSD plot of the wild type protein structure can be viewed in Figure 2.

#### 3.4.2. RMSD for Mutant Protein Structure

In the mutant structure the average RMSD trajectory value ranges between 0.14 nm and 0.42 nm. The mutant protein structure is seen to be almost constant till the end. Slight deviations in this protein structure can be noticed at 15000 ps, 17000 ps, and 22000 ps. A slight rise in the RMSD value of this structure is noticed at 48500 ps after which the RMSD trajectory value of this protein structure is seen to be constant till the end of the simulation time period.

The RMSD trajectory is used to predict the stability of the protein. Higher RMSD value implies low stability of the protein structure. From the RMSD results of both wild and mutant protein structures, the mutant protein structure shows more stability when compared to the wild type protein structure. Both the trajectories show almost the same trajectory value till 12000 ps after which the mutant structure has become stable making the wild type structure comparatively unstable [11, 38, 39]. The result of stability obtained in this study is similar to the experimental evidence of T118M causing CMT 1A [11].

### 3.5. RMSF

**Figure 3:**
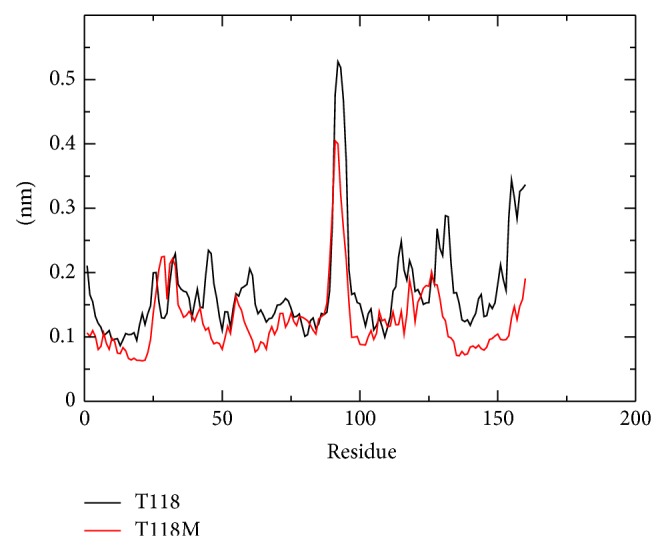
RMSF of the backbone CAs of C*α* atoms of wild type (black) and mutant (red) PMP22 protein motor domain versus time at 300 K.

With the aim of determining whether the mutation affects the dynamic behavior of residues, the RMSF values of wild type and mutant structures were compiled (Figure 3). The RMSF with respect to the average MD simulation conformation is used as a mean describing flexibility differences among residues. The backbone RMSF of each residue of wild type and mutant class PMP22 are calculated in order to analyze the flexibility of backbone structure. Higher RMSF value shows more flexible movements whereas low RMSF value shows limited movements during simulation in relation to its average position.

#### 3.5.1. RMSF for Wild Type Protein Structure

The RMSF range of the wild type protein structure is between 0.09 nm and 0.52 nm. High fluctuation in this protein structure can be seen throughout the simulation time period. High fluctuation can be noticed at residues positions 26, 33, 45, 60, 92, 114, 118, 131, and 155. The highest RMSF fluctuation is at residue number 92 with a fluctuation of 0.52 nm.

#### 3.5.2. RMSF of Mutant Protein Structure

The RMSF range of the mutant protein structure is between 0.06 nm and 0.39 nm. From the graph we can clearly notice that there is an overall decrease in the fluctuation of the mutant protein residues when compared to the wild type structure. High fluctuation can be noticed in residues numbers 29, 32, 55, 91, 92, 118, 126, and 160. The highest residual fluctuation in this protein structure can be noticed at residue number 92 with the fluctuation of 0.39 nm.

From the RMSF plot in Figure 3 we can notice that the wild type protein structure shows very high fluctuations at almost all residual positions when compared to the mutant protein structure. The terminal residues of both the protein structures show high fluctuations. The RMSF result shows that the mutant protein structure is more stable than the wild type structure. Thus, the result of T118M mutation in the PMP22 gene causes an overall increase in stability of the mutant type protein structure when compared to the wild type protein structure. This can be correlated with CMT related disorder [11].

### 3.6. Radius of Gyration

We performed Rg in order to understand the levels of compaction of the native and mutant class PMP22. The Rg is generally defined as the mass weighted root mean square distance of a collection of atoms from their common center of mass. Hence, this analysis gives us the overall dimensions of the protein.

#### 3.6.1. Rg for Wild Type Protein Structure

The Rg range of the wild type protein structure is between 1.67 nm and 1.77 nm. At 5500 ps, we can see an increase in the Rg value to 1.76 nm. From 5500 ps till 14500 ps, there is a continuous decrease in the Rg value of this protein structure. A decrease in the Rg value can be noticed at 22200 ps and 24700 ps after which the Rg value of this protein structure is almost constant till the end.

#### 3.6.2. Rg for Mutant Protein Structure

The Rg range for mutant protein structure is between 1.63 nm and 1.76 nm. A decrease in the Rg value of this protein structure is clearly noticed from 13000 to 16000 ps and 27000 to 32500 ps. A very great increase in the Rg value for this protein structure can be noticed from 44000 ps till the end of the simulation time period.

**Figure 4:**
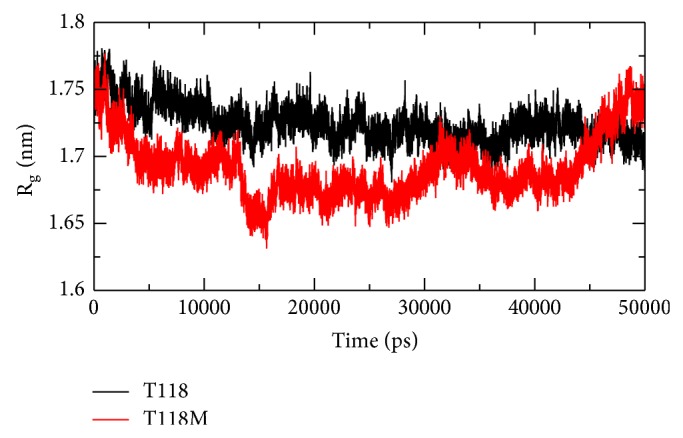
Radius of gyration of C*α* atoms of wild type (black) and mutant (red) PMP22 protein motor domain versus time at 300 K.

From the Rg plot we can say that the wild type protein structure has a greater Rg value when compared to the wild type protein structure making the wild type protein structure relatively unstable when compared to the mutant protein structure. This result can be supported from the PCA plot obtained. The Rg plot can be noticed in Figure 4.

### 3.7. Hydrogen Bonds

H bonds play a vital role in molecular recognition and the overall stability of the protein structure. Intermolecular H bond is analyzed for the wild type and mutant structures of PMP22 protein during the simulation period.

#### 3.7.1. H Bond for Wild Type Protein Structure

From the analysis, the differences in protein-solvent interactions are observed in the wild type and mutant structures. The number of hydrogen bonds in the wild type protein structure is lesser than the mutant protein structure throughout the simulation time period. The lesser number of hydrogen bonds in wild protein structure makes it relatively unstable.

#### 3.7.2. H Bond for Mutant Protein Structure

The mutant protein structure has higher number of hydrogen bonds when compared to the wild type protein structure throughout the simulation time period. At 16000 ps, 24500 ps, and 36500 ps we can see dense number of hydrogen bonds in this protein structure.

**Figure 5:**
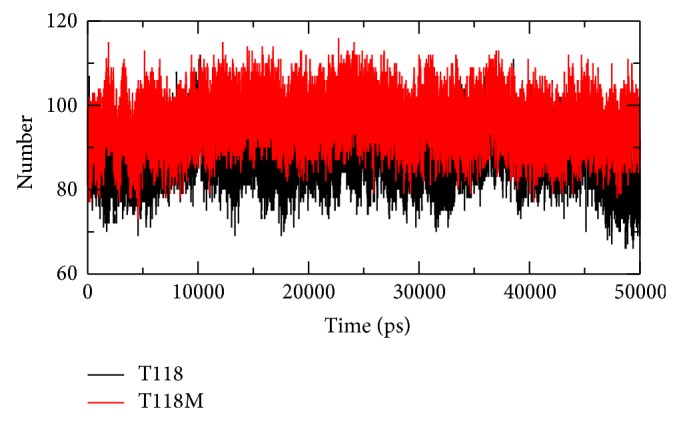
Intermolecular hydrogen bonds in wild type (black) and mutant (red) PMP22 protein versus time at 300 K.

We know that the number of hydrogen bonds in a protein structure determines its stability. Here, the mutant protein structure is relatively stable compared to the wild type protein structure due to higher number of hydrogen bonds. This plot can be viewed in Figure 5.

### 3.8. Solvent Accessible Surface Area

SASA is to understand the solvent accessibility of the wild type and mutant PMP22 structures. SASA plot accounts for bimolecular surface area that is assessable to solvent molecules. The rise in SASA value denotes relative expansion.

#### 3.8.1. SASA for Wild Type Protein Structure

The SASA range of this protein structure lies between 59 nm^2^ and 74 nm^2^. At 11500 ps we can see a SASA value of 72 nm^2^. There is a drop in the SASA value of this protein structure between 23000 ps and 30500 ps. From 31000 ps to 40000 ps we can see noticeable deflection in the SASA value. At 40800 ps, we can see the lowest SASA value of 59 nm^2^. From 44000 ps till the end of the simulation time period the SASA value of this protein structure is constant. The amount of the PMP22 protein is shown to decrease in a previously performed experimental study which is exactly correlated with our SASA results [11].

#### 3.8.2. SASA for Mutant Protein Structure

**Figure 6:**
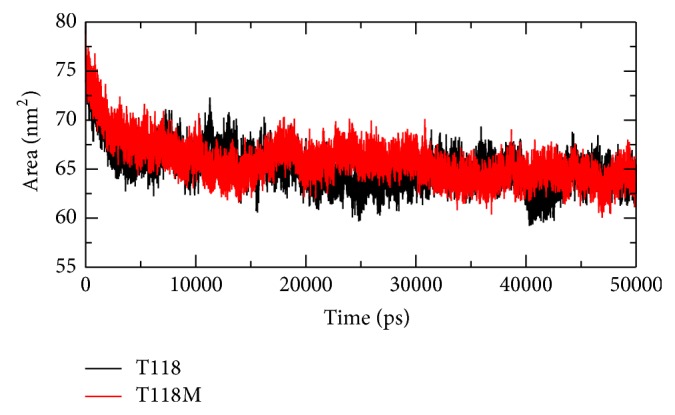
Solvent accessible surface area (SASA) of wild type (black) and mutant (red) PMP22 protein versus time at 300 K.

The SASA range of this protein structure lies between 60 nm^2^ and 77 nm^2^. At 17500 ps, we can see an increase of the SASA value to 70 nm^2^ after which it is almost constant till the end of the simulation time period. The resulting of the mutation showed decrease in the amount of protein which says that it shows a decrease in the solvent accessible surface area that results in an increase in stability of the protein. The SASA plot can be viewed in Figure 6. From the graph, we can see that the SASA value of the mutant protein structure is overall greater than the wild type protein structure making the mutant structure more stable [11].

### 3.9. Principle Component Analysis

PCA is a technique that can sort out all of the locally restricted fluctuations and vibrational motions. An improved outlook of the dynamical mechanical properties of the investigated method has been obtained by using essential dynamics (ED) analysis. The large scale collective motions of the wild type structure and mutant structure using ED analysis are determined to further support our MD simulation results. The confined fluctuation and structural motion of the wild type and mutant structures are determined using ED analysis. The dynamics of all the structures are best achieved via characterization of their phase space behavior. The eigenvectors of the covariance matrix are called its principle components. The change of particular trajectory along each eigenvector is obtained by this projection. The spectrum of the corresponding eigenvalues represents that the structural motion of the system is basically confined within the first two eigenvectors. The projections of trajectories obtained at 300 K on the first two principal components (PC1 and PC2) showed the structure motion of the wild type and mutant proteins in phase space. More distribution of dots indicates more conformational changes in protein structure. The internal motions of mutant PMP22 represented a subspace whose dimension is much bigger than the wild type protein. It is revealed that the concerted motions increased in the mutant PMP22 and are in agreement with MD analysis.

**Figure 7:**
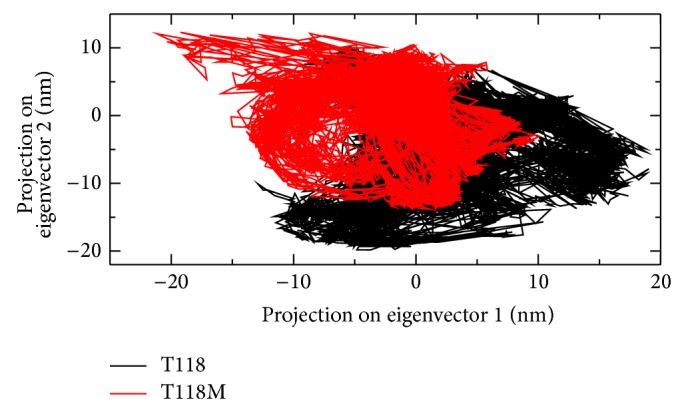
Projection of the motion of the wild type (black) and mutant (red) PMP22 proteins in phase space along the first two principal eigenvectors at 300 K.

Firstly, the clusters are well defined in mutant than wild type. Secondly, wild type covered a larger region of phase space along both PC1 and PC2 plane than mutant and it is depicted in Figure 7. Our observation thus corroborates with the idea of higher flexibility of wild type than mutant at 300 K. The overall flexibility of two proteins is also calculated by using the trace of the diagonalized covariance matrix of the C*α* atomic positional fluctuations. We have obtained the following values for wild type protein which is 5.46711 (nm^2^) and for the mutant protein which is 3.1486 (nm^2^) again confirming the overall increased flexibility of wild type than mutant at 300 K. Our results reported that the substitution of T118M in PMP22 has increased the structural stability [11].

**Figure 8:**
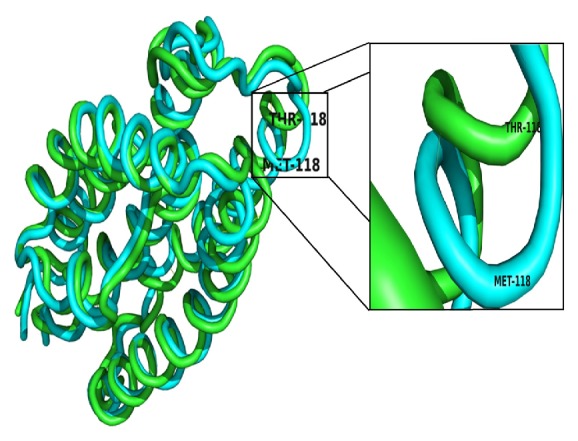
Superimposed backbone protein structures of wild type and mutant T118M after 50 ns.

**Figure 9:**
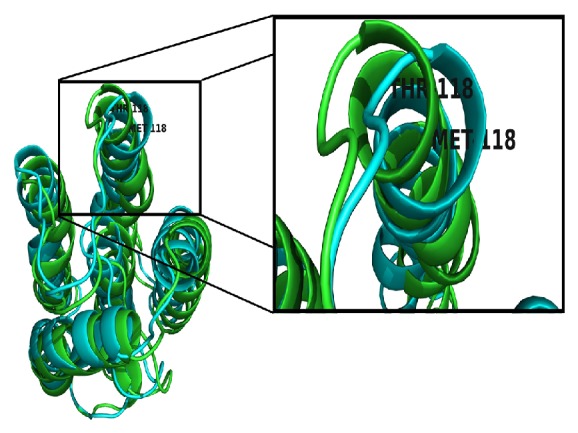
Superimposed protein structures of wild type and mutant T118M after 50 ns.

After molecular dynamics simulation, we superimposed the two protein structures obtained after MD simulation. There is an observable difference between the two structures. The superimposed backbone protein structure can be viewed in Figure 8. In the mutant protein structure, threonine which is a polar, nonaromatic hydroxyl and is hydrophilic in nature is replaced by methionine. Methionine is a sulfur containing, nonpolar hydrophobic amino acid. These two amino acids have different biochemical properties. Hence the mutation results in the changes in structure after MD simulation. The superimposed protein structures can be seen in Figure 9.

## 4. Discussion

The correlation between the genotype and phenotype is generally explained by using genome sequencing and its analysis. The occurrence of disease is possible to be predicted by observations on the effect of point mutations at the protein level. These observations can be done by using advanced methodology in computational biology and the consequence of deleterious mutations can be predicted. The computational study to determine the genotypic-phenotypic association and possible pathogenic consequences at disease level is not carried out to higher accuracy level. To examine the structural consequence and the stability of the above predicted CMT1A associated nsSNP we performed molecular dynamics simulation of the prioritized mutant and the wild type PMP22 protein. This study provided us a detailed idea about the structural aspects of T118M PMP22 mutation and its effect on CMT1A and it also gave clues to carry out computational studies using the computational platforms to predict CMT1A associated nsSNPs with a relatively higher accuracy level. A total of 26 SNPs got from dbSNP were selected for this study which were subjected to SIFT, PolyPhen2, PMUT, PhD-SNP, SNP&GO, and MutPred to examine and predict the CMT1A associated mutations with a relatively higher accuracy. Initially, out of the 26 inputs, 11 were predicted to be deleterious using SIFT, PolyPhen2, and PMUT (Table 1). Further, 10 (R157W, L16P, T118M, M69K, H12Q, G150C, S22F, W28R, D37V, and Y136S) were predicted to be disease associated using PhD-SNP and SNP&GO (Table 2). T118M with a (*g*) score of 0.948 was screened to be highly deleterious using the MutPred server (Table 3). Since T118M showed high confident hypothesis, we carried out MD simulation for this mutation. We highlighted the RMSF of backbone carbon by trajectory analysis obtained through the performed MD simulation. H bond analyses were performed to understand the flexibility behavior of residues. We also calculated the RMSD for all the C*α* atoms from the initial structure, which were considered a central criterion to measure the convergence of the protein system concerned. In Figure 2, both the trajectories show almost the same value till 12000 ps after which the mutant structure has become stable making the wild type structure comparatively unstable. The Rg is known to be defined as the mass-weighted root mean square distance of a collection of atoms from their common center of mass. Hence, this analysis gives us an insight into the overall dimensions of the protein. The plot of radius of gyration of C*α* atoms of the protein versus time at 300 K is shown in Figure 4. We observed a slight rise in radius of gyration in wild type structure as compared to the mutant which further supported our hypothesis. The Rg results can be correlated with the PCA results obtained. Mutant structure exhibited more flexibility compared to wild type. To investigate the flexible behavior of binding residues, we plotted the RMSF of C*α* atoms between the residues. Mutant structure residues were found to exhibit small flexibility as compared to wild type. Intermolecular H bond was calculated for wild type and mutant structure during the simulation time. Notable differences in protein-solvent interactions were evident in wild type and mutant structure in Figure 5. More intermolecular H bond in the mutant structure might help to maintain its rigidity while a slightly higher tendency of the wild type to be involved in hydrogen bonding with solvent makes it more flexible. On the basis of RMSF observation and H bond analysis, it is confirmed that the occurrence of the mutation led to a less flexible conformation due to the formation of higher number of hydrogen bonds. On the basis of the molecular dynamics simulation results we got a reverse conclusion regarding the flexibility and the stability of the wild type and mutant structure [38]. The projection of trajectories obtained at 300 K onto the first two principal components (PC1, PC2) showed the motion of two proteins in phase space. On these projections, we saw clusters of stable states. Two features were very apparent from these plots. We have obtained the following values for wild type protein which was 5.46711 (nm^2^) and for the mutant protein which was 3.1486 (nm^2^) again confirming the overall increased flexibility of wild type than mutant at 300 K. The T118M mutant protein was seen to be more stable when compared to the wild type protein structure which was correlated with published literature on PMP22 protein. The T118M mutant protein structure exhibited loss of sheet even while being more stable than the wild type protein. From this, we can say that the increase in stability of the mutant protein structure leads to CMT1A which has been proved in previous experimental study, which suggests that the T118M protein functions normally but results in the reduction of the protein [11]. This is proved in the Rg and PCA graphs in our study.

## 5. Conclusion

CMT neuropathies are traditionally called hereditary motor and sensory neuropathies (HMSNs). CMT1A is associated with the mutation in the PMP22 gene. One or the other point mutation in PMP22 can result in the disease phenotype. This gene is seen to change its behavior at multiple point mutation positions. Change in function is observed which has been proved to cause CMT1A in several cases. Any disturbance in these positions leads to CMT1A which is proved previously through experimental studies. The most common mutation in this gene that causes CMT1A is T118M. This mutation is found to cause CMT1A in several cases. In this study we proved the gain of stability in the PMP22 gene due to T118M mutation. This gain in stability leads to CMT1A. We also showed the conformational changes between the two structures as evidence of structural changes in the PMP22 protein due to the mutation T118M. A clear gain of stability is seen in the RMSD and Rg plots of this study. The gain in the number of H bonds also adds evidence to the conclusion. We assume this to be the first computational report on the stability of PMP22 mutation in CMT1A; it can be useful for further research.

## Figures and Tables

**Table 1:** nsSNPs of PMP22 gene analyzed by three computational methods: PolyPhen 2.0, SIFT, and PMUT.

SNP ID		SIFT			PolyPhen2		PMUT
	Mutation	Score	Prediction	PSIC	Prediction	Score	Prediction
rs28936682	R157W	0.00	Deleterious	1.000	Probably damaging	0.9963	Pathological
rs104894617	L16P	0.03	Deleterious	0.928	Possibly damaging	0.7069	Pathological
rs104894618	S79C	0.02	Deleterious	0.992	Probably damaging	0.5895	Pathological
rs104894619	T118M	0.00	Deleterious	1.000	Probably damaging	0.6326	Pathological
rs104894620	M69K	0.01	Deleterious	0.994	Probably damaging	0.8504	Pathological
rs104894621	S72L	0.00	Deleterious	0.999	Probably damaging	0.4180	Neutral
rs104894622	H12Q	0.01	Deleterious	0.546	Possibly damaging	0.5511	Pathological
rs104894623	A67T	0.00	Deleterious	1.000	Probably damaging	0.1614	Neutral
rs104894624	G150C	0.02	Deleterious	1.000	Probably damaging	0.9706	Pathological
rs104894625	S22F	0.00	Deleterious	0.960	Probably damaging	0.8126	Pathological
rs104894626	W28R	0.00	Deleterious	1.000	Probably damaging	0.9771	Pathological
rs104894627	D37V	0.01	Deleterious	1.000	Probably damaging	0.6415	Pathological
rs11545341	Q86K	0.12	Tolerated	0.997	Probably damaging	0.5282	Pathological
rs112232836	K92R	0.27	Tolerated	0.998	Probably damaging	0.0661	Neutral
rs112651887	T44A	0.51	Tolerated	0.001	Benign	0.0557	Neutral
rs138515303	I9V	0.35	Tolerated	0.041	Benign	0.0326	Neutral
rs140763467	G133S	0.06	Tolerated	1.000	Probably damaging	0.8228	Pathological
rs141094419	A135T	0.09	Tolerated	0.001	Benign	0.6424	Pathological
rs147114400	L90V	0.08	Tolerated	0.476	Possibly damaging	0.2900	Neutral
rs148822354	A115V	1.00	Tolerated	0.005	Benign	0.4346	Neutral
rs189205303	T89N	0.11	Tolerated	0.997	Probably damaging	0.4841	Neutral
rs201255121	P144T	0.31	Tolerated	1.000	Probably damaging	0.5560	Pathological
rs368223794	A106T	0.30	Tolerated	0.865	Possibly damaging	0.2658	Neutral
rs368908933	H51R	0.33	Tolerated	0.000	Benign	0.1470	Neutral
rs373322590	N32S	0.47	Tolerated	0.011	Benign	0.1235	Neutral
rs375449671	Y136S	0.00	Deleterious	0.636	Possibly damaging	0.7302	Pathological

**Table 2:** The disease associated SNPs are predicted from PHDsnp and SNP&GO.

SNP ID	Mutations	PHDsnp		SNP&GO	
		RI score	Effect	Score	Effect
rs28936682	R157W	8	Disease	8	Disease
rs104894617	L16P	6	Disease	6	Disease
rs104894618	S79C	6	Disease	0	Neutral
rs104894619	T118M	7	Disease	6	Disease
rs104894620	M69K	9	Disease	8	Disease
rs104894622	H12Q	6	Disease	7	Disease
rs104894624	G150C	6	Disease	8	Disease
rs104894625	S22F	6	Disease	7	Disease
rs104894626	W28R	9	Disease	9	Disease
rs104894627	D37V	9	Disease	8	Disease
rs375449671	Y136S	7	Disease	8	Disease

**Table 3:** The *G* score, *P* score, molecular variations, and prediction reliability calculated from MutPred server.

SNP ID	Mutations	*G* score	*P* score	MutPred	Prediction reliability
rs28936682	R157W	0.887	0.0022	Loss of disorder	Very confident hypotheses
rs104894617	L16P	0.939	0.0826	Loss of stability	No reliable inference
rs104894618	S79C	0.936	0.0098	Gain of catalytic residue	Very confident hypotheses
rs104894619	T118M	0.948	0.0045	Loss of sheet	Very confident hypotheses
rs104894620	M69K	0.943	0.0325	Loss of stability	Confident hypotheses
rs104894622	H12Q	0.894	0.0865	Gain of catalytic residue	No reliable inference
rs104894625	S22F	0.873	0.0028	Gain of disorder	No reliable inference
rs104894624	G150C	0.966	0.0510	Loss of glycosylation at S149	No reliable inference
rs104894626	W28R	0.951	0.0344	Loss of sheet	Confident hypotheses
rs104894627	D37V	0.957	0.0521	Loss of disorder	No reliable inference
rs375449671	Y136S	0.758	0.0130	Loss of stability	Actionable hypotheses

## Abbreviations

CMT1A: Charcot Marie Tooth, type 1A
PMP22: Peripheral myelin protein 22
nsSNPs: Nonsynonymous single nucleotide polymorphisms
MDS: Molecular dynamics simulation
RMSD: Root-mean-square deviation
Rg: Radius of gyration
SASA: Solvent accessible surface area
RMSF: Root-mean-square fluctuation
NH bonds: Number of hydrogen bonds.
